# Mean‐stress sensitivity of an ultrahigh‐strength steel under uniaxial and torsional high and very high cycle fatigue loading

**DOI:** 10.1111/ffe.13767

**Published:** 2022-06-27

**Authors:** Bernd M. Schönbauer, Sumit Ghosh, Ulrike Karr, Sakari Pallaspuro, Jukka Kömi, Tero Frondelius, Herwig Mayer

**Affiliations:** ^1^ Institute of Physics and Materials Science University of Natural Resources and Life Sciences Vienna (BOKU) Austria; ^2^ Materials and Mechanical Engineering, Centre for Advanced Steels Research University of Oulu Oulu Finland; ^3^ R&D and Engineering Wärtsilä Vaasa Finland

**Keywords:** $\sqrt{\text{area}}$‐parameter model, fatigue limit, load ratio, thermomechanically processed ultrahigh‐strength steel, torsional fatigue, ultrasonic fatigue

## Abstract

The influence of load ratio on the high and very high cycle fatigue (VHCF) strength of Ck45M steel processed by thermomechanical rolling integrated direct quenching was investigated. Ultrasonic fatigue tests were performed under uniaxial and torsional loading at load ratios of *R* = −1, 0.05, 0.3, and 0.5 with smooth specimens and specimens containing artificially introduced defects. Up to 2 × 10^5^ cycles, failure originated from surface aluminate inclusions and pits under both loading conditions. The prevailing fracture mechanisms in the VHCF regime were interior crack initiation under uniaxial loading and surface shear crack initiation under torsional loading. The mean‐stress sensitivity and the fatigue strength were evaluated using fracture mechanics approaches. Equal fatigue limits for uniaxial and torsional loading were determined considering the size of crack initiating defects and the appropriate threshold condition for Mode‐I crack growth. Furthermore, the mean‐stress sensitivity is independent of loading condition and can be expressed by $\sigma_{w}\left(R\right)={\left.\sigma_{w}\right|}_{R=-1}\cdot {\left(\frac{1-R}{2}\right)}^{0.63}$ and $\tau_{w}\left(R\right)={\left.\tau_{w}\right|}_{R=-1}\cdot {\left(\frac{1-R}{2}\right)}^{0.63}$.

## Highlights

1. The uniaxial and torsional fatigue limit is equal for ultrahigh‐strength steels.
2. The mean‐stress sensitivity is identical for uniaxial and torsional loading.
3. The $\sqrt{\text{area}}$‐parameter model predicts the fatigue limit in the presence of small defects.
4. Ambient air is a chemically active environment for ultrahigh‐strength Ck45M steel.

## 1 INTRODUCTION

The effect of load ratio on the fatigue strength has been extensively studied for various materials. However, most investigations are limited to the low and high cycle fatigue (HCF) regime, and the majority of experiments were conducted under uniaxial loading. Several studies have been performed during the last decades with focus on the influence of mean tensile stresses on the very high cycle fatigue (VHCF) properties.^1^, ^2^, ^3^, ^4^, ^5^, ^6^, ^7^, ^8^, ^9^, ^10^, ^11^ In the VHCF regime, fracture of steels is typically associated with crack initiation from the interior,^10^, ^12^, ^13^, ^14^ but the mean stress can play a significant role for the prevailing fracture mechanism. Recent investigations that were performed to study the effect of superimposed shear stresses under torsional loading, for example, have shown that interior crack initiation under reversed torsional loading is rarely observed while this fracture mode is prevalent in VHCF at positive load ratios, *R* ≥ 0.^15^ Also, under uniaxial loading, some investigations revealed that fracture from the interior at lifetimes exceeding 10^8^ cycles can only be observed at positive *R* ratios.^4^, ^5^

The fatigue strength of high‐ and ultrahigh‐strength steels is typically correlated with the size of the largest—or most detrimental—inherent flaw. Due to the material's low defect tolerance, even flaws with sizes of a few microns lead to a decrease of the fatigue limit. Most frequently observed origins of fracture are nonmetallic inclusions such as aluminates, nitrides, or sulfates. Further, soft phases such as bainite or delta‐ferrite in a martensitic matrix, grain boundaries or any other inherent defects large enough to initiate a fatigue crack may affect the fatigue strength.^16^, ^17^ Such small defects can be generally considered to behave similarly to (small) cracks. Consequently, fracture mechanics principles can be applied to predict the fatigue limit by relating the stress intensity factor range of a defect with the appropriate threshold value. Probably the most widely used prediction approach used to estimate the fatigue limit in the presence of small defects is the so‐called $\sqrt{\text{area}}$‐parameter model proposed by Murakami and Endo.^18^, ^19^ This simple approach allows to predict the fatigue limit based on the size‐dependent threshold stress intensity factor that is calculated by only two parameters: the material's hardness and the square root of the projection area of the defect perpendicular to the major principal stress direction, $\sqrt{\text{area}}$.

Considering the fact that the fatigue limit of a test specimen depends on the largest detrimental defect in its test volume, it is clear that the size of specimens has an influence on the results: From a statistical point of view, the size of the largest defect increases with increasing the specimen's volume subjected to nominal stress.^16^, ^20^ This is especially relevant when laboratory test results are adapted to component design. Furthermore, depending on the statistical distribution of defects, their maximum sizes can significantly vary from specimen to specimen. An indication for significantly different sizes of detrimental defects in a test series is a huge scatter in *S*–*N* data. This important issue is often neglected when the mean‐stress sensitivity of a material is evaluated using laboratory test results obtained with a limited number of specimens. For example, the fatigue limit determined at different load ratios may be significantly affect by outliers, e.g., huge inclusions that can be only found in few specimens. Introducing small artificial defects of uniform size—which must be larger than the expected size of inherent defects—into test specimens is an appropriate way to avoid this problem.^11^, ^16^, ^21^ However, as presented in a recent study with martensitic stainless steel 17‐4PH^22^ and successfully applied to other high‐strength steels,^11^, ^23^ the size of defects generating fracture in different test specimens can also be incorporated in the assessment of the mean‐stress sensitivity. This method will be also applied to evaluate the *R*‐ratio dependency of the ultrahigh‐strength steel investigated in the present work.

Further focus of this study is on changes in failure mechanisms depending on fatigue lifetime, mean stress and loading condition. Comprehensive fatigue tests up to more than 10^10^ cycles were performed under uniaxial as well as torsion loading at different load ratios between *R* = −1 and 0.5 using the ultrasonic‐fatigue testing technique. A previous investigation with the thermomechanically processed (TP) and subsequently direct quenched (DQ) Ck45M steel focused on the fully reversed tension–compression fatigue properties in the HCF and VHCF regime.^24^ *S*–*N* tests as well as fatigue crack growth rate measurements in the near‐threshold regime revealed clear environmental influences in ambient air, resulting in fracture that originates from the surface even in the VHCF regime. In the present study, further insights are obtained in this context with respect to the effect of mean tensile and shear loads under uniaxial and torsion VHCF loading, respectively.

## 2 MATERIAL AND EXPERIMENTAL PROCEDURE

The investigations were performed with TMP and subsequently DQ Ck45M/AISI 1045 steel (TMP‐DQ) that has been already used in a previous study on the tension–compression VHCF strength.^24^ This Ca‐treated steel in its as‐received condition is typically used in machined parts in mechanical engineering and automotive components exposed to cyclic loading. It is used also in hardened condition, typically produced via case, flame, or induction hardening, with an approximate surface hardness up to 60 HRC, which is only slightly lower than what is achieved with TMP‐DQ processing. As TMP‐DQ produces homogeneous material properties, it is well suited for studying the fatigue behavior of these hardened machine elements.

After soaking a block with a thickness of 60 mm at 1150°C for 1 h, the material was thermomechanically rolled in two stages: In the first stage, hot rolling was conducted in three passes above the no‐recrystallization temperature (1080 ± 10°C, 1050 ± 10°C, and 1020 ± 10°C) resulting in a thickness of ~31.5 mm with ~0.2 strain/pass. The second stage comprised three further passes between the no‐crystallization temperature and the ferrite start temperature (920 ± 10°C, 860 ± 10°C, and 830 ± 10°C). Immediately after hot rolling, the block with a final thickness of 15.5 mm (~0.2 strain/pass) was direct quenched in water to room temperature (~60°C/s). The resulting microstructure contains highly dislocated lath martensite and finely divided, film‐like interlath retained austenite (RA) with a volume fraction of martensite and RA of approximately 96% and 4%, respectively.

**FIGURE 1:**
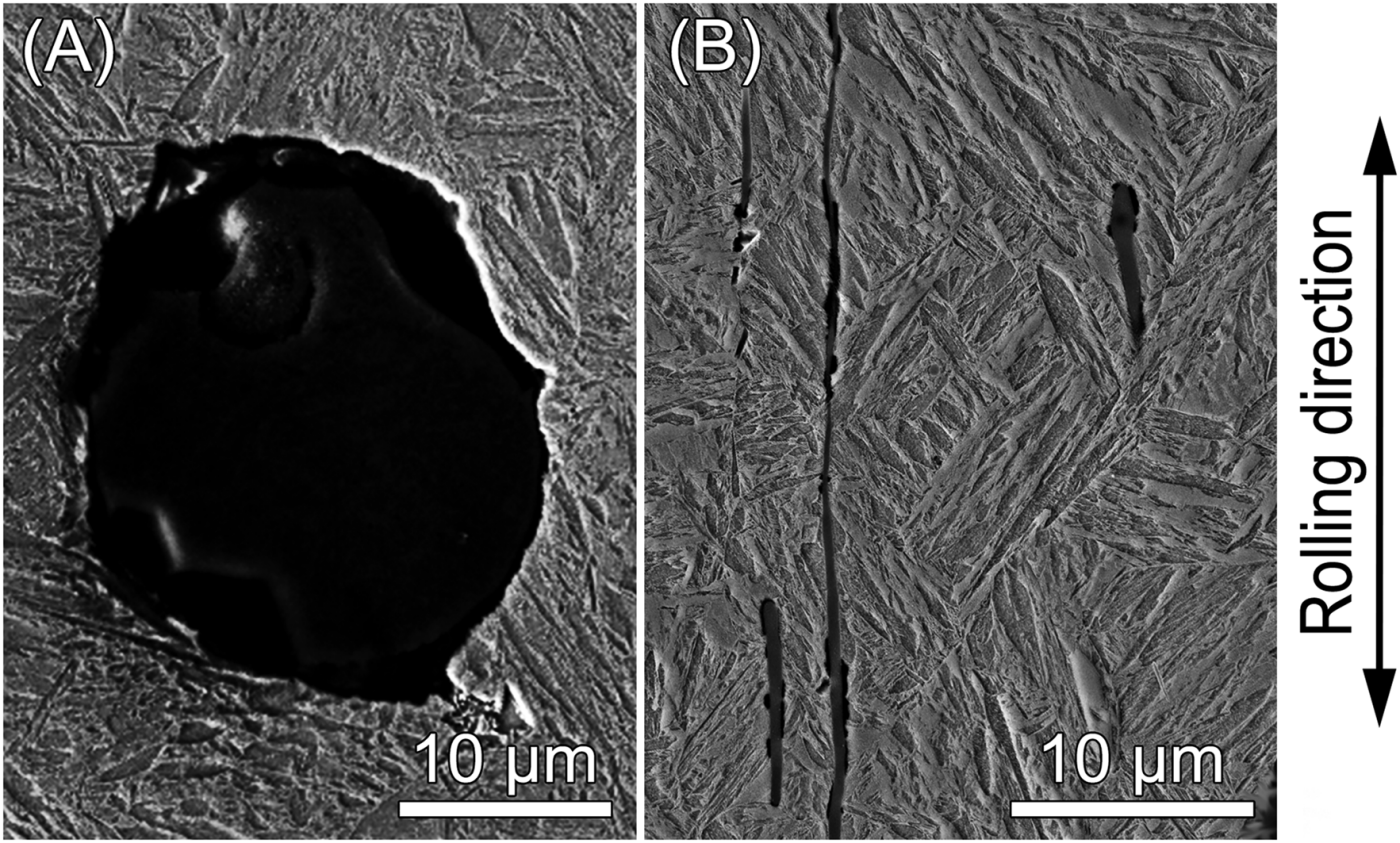
Micrographs of Ck45M steel (TMP‐DQ) with (A) Al_2_O_3_–CaO and (B) MnS inclusion

Secondary electron micrographs, acquired with field‐emission scanning electron microscopy (FE‐SEM), of the investigated Ck45M steel (TMP‐DQ) are shown in Figure 1. Energy‐dispersive X‐ray spectroscopy (EDX) of inclusions identified globular aluminum/calcium oxide (Al_2_O_3_–CaO) inclusions encapsulated in calcium sulfides (Figure 1A) and manganese sulfide (MnS) stringers elongated in rolling direction (Figure 1B).

The chemical composition and the mechanical properties are given in Tables 1 and 2, respectively. More details on the processing and the microstructural features can be found in Schönbauer et al.^24^

**TABLE 1:** Chemical composition in weight %

C	Mn	Si	Pl	S	Cr	Cu	B	Mo
0.440	0.667	0.294	0.009	0.036	0.218	0.221	0.0002	0.034

**TABLE 2:** Mechanical properties

0.2% proof stress (MPa)	Tensile strength(MPa)	Elongation(%)	Vickers hardness(kgf/mm^2^)
1613	2439	3	717

**FIGURE 2:**
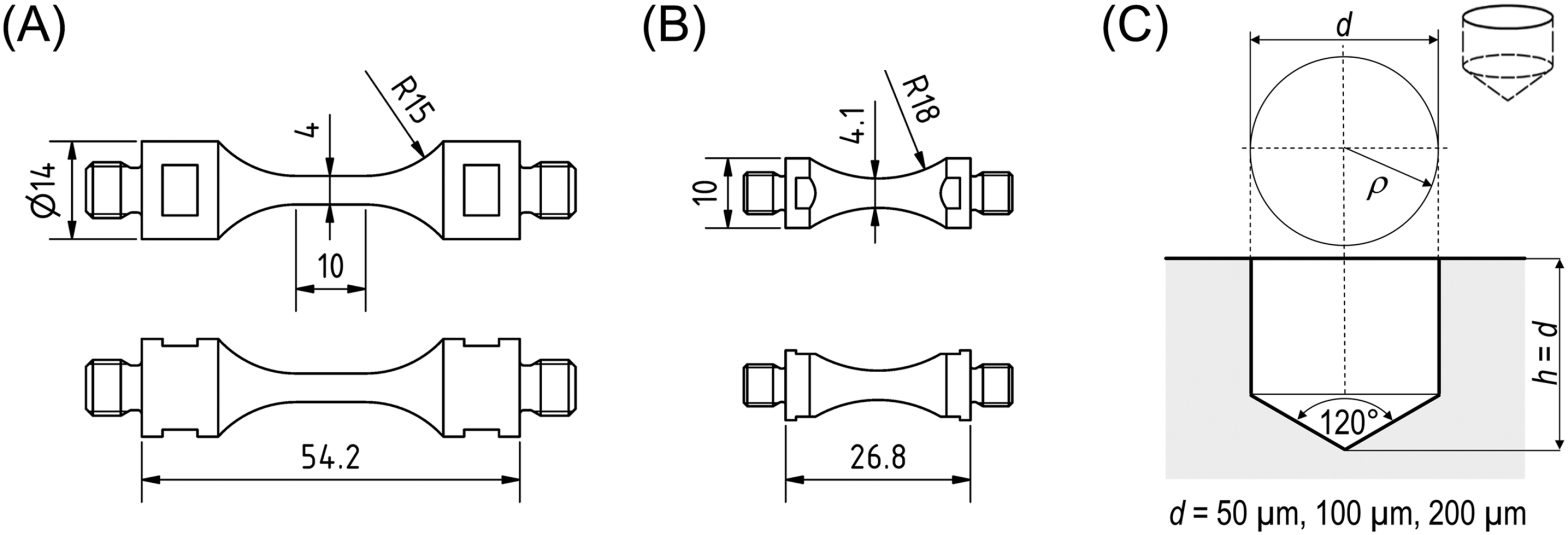
Specimen shapes for (A) uniaxial and (B) torsional fatigue tests (in mm). (C) Geometry of artificial defects (drilled hole)

Test specimens were machined with their length axes in rolling direction. In Figure 2, specimen geometries for uniaxial (A) and torsional (B) testing are shown. A surface layer of around 100 μm was removed in the gauge section of all specimens by grinding (emery paper up to #2000) and electropolishing with the aim to remove any residual stresses introduced during specimen machining.

In order to introduce well‐defined defects as fatigue crack initiation sites, small holes were drilled into the surface of some uniaxial and torsional specimens using a high‐precision drill press. Hole diameters, *d*, and depths, *h*, (with *h* = *d*) were 50, 100, and 200 μm. The geometry of holes can be seen in Figure 2C.

*S–N* tests were performed employing the ultrasonic fatigue testing equipment developed at BOKU. Specimens are cycled in resonance at about 19 kHz, and a closed‐loop control with an induction coil that measures the vibration of one specimen's end as feedback ensures high accuracy of the vibration amplitude. Both uniaxial and torsional tests were conducted at load ratios of *R* = −1, 0.05, 0.3, and 0.5. If specimens are fixed with only one end into to the resonance system (with a thread as shown in Figure 2), cyclic loading is fully reversed, i.e., the load ratio is *R* = −1. Mean loads can be superimposed by fixing an elongation rod at the other end of the specimen and applying a tensile or shear load at the elongation rod's vibration node. A detailed description of the testing technique and applications of ultrasonic fatigue testing is given in Mayer.^25^ Compressed‐air cooling and pulsed loading, i.e., intermittent pauses during high‐frequency testing, served to prevent self‐heating of specimens. Fatigue testing was conducted in ambient air at approximately 23°C and 50% relative humidity.

## 3 RESULTS AND DISCUSSION

### 3.1 *S–N* tests results

#### 3.1.1 Smooth specimens

**FIGURE 3:**
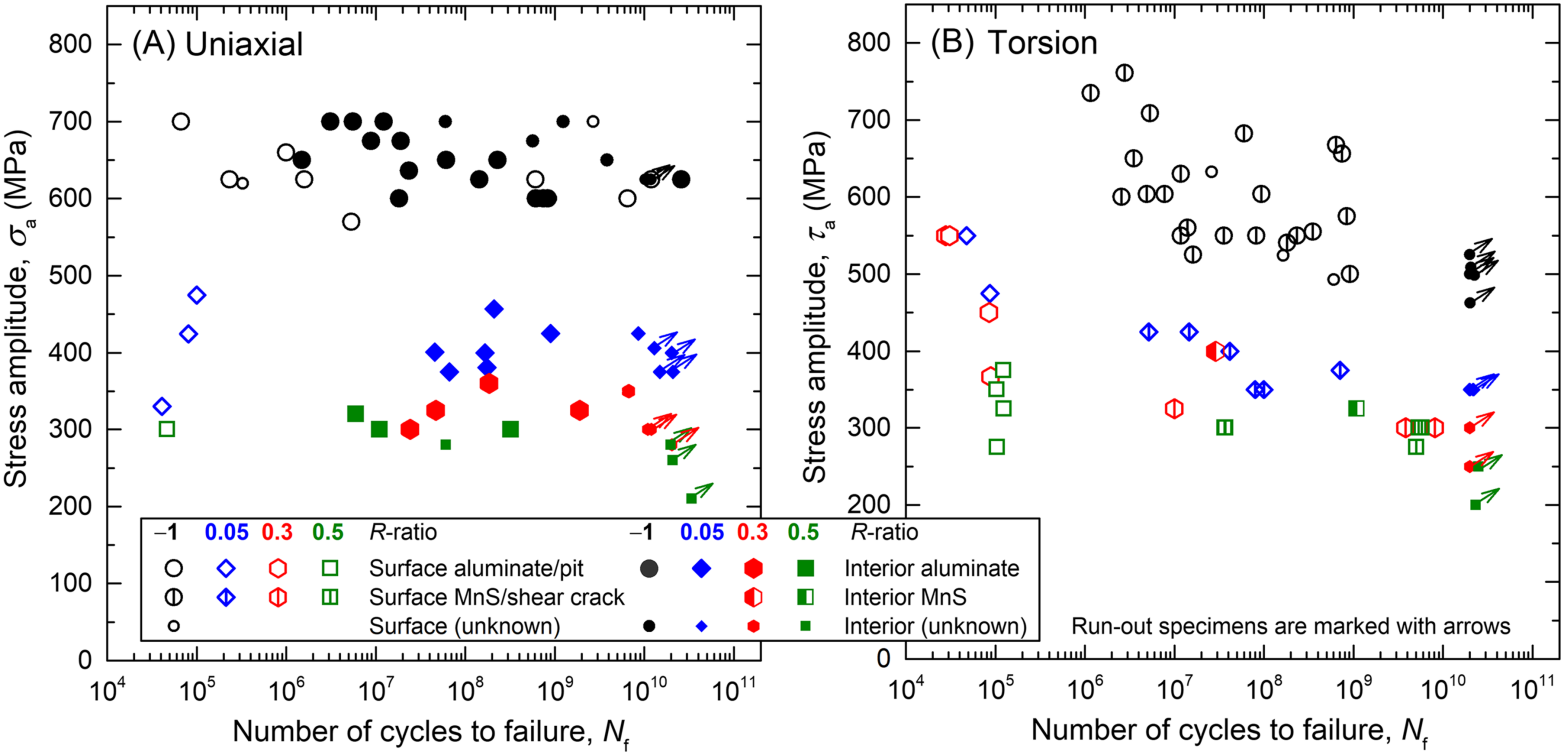
*S–N* diagrams for (A) uniaxial and (B) torsional test results at different load ratios(data obtained under uniaxial loading at *R* = −1 were already reported in Schönbauer et al.^24^) [Colour figure can be viewed at wileyonlinelibrary.com]

The results of uniaxial fatigue tests are plotted in Figure 3A. There is a huge scatter of fatigue lifetimes, albeit a clear decrease in bearable stress amplitudes with increasing mean stress is observable. Tests were performed up to at least 10^10^ cycles, but failure even occurred after 2.56 × 10^10^ cycles. Hence, no fatigue limit could be determined. The origin of fatigue failure for the majority of test specimens was a nonmetallic inclusion located at the surface or in the interior (see Section 3.2). If failure occurred within 10^6^ cycles, crack initiation was solely at the surface. For longer lifetimes, both surface and interior crack initiation were found—even in the VHCF regime. This is rather unusual for steels since failure in the VHCF regime is typically associated with interior fracture. However, the corrosion resistance of steels tends to decrease with increasing strength, and environmental effects may influence crack initiation and propagation even in ambient air. A detailed discussion on VHCF failure from the surface at fully reversed loading has been already given in Schönbauer et al.^24^

Fatigue test results for cyclic torsional loading are shown in Figure 3B. Under fully reversed loading, torsional fatigue tests led to lower fatigue strengths than uniaxial fatigue tests. At positive load ratios, in contrast, no clear difference can be discerned. Below 2 × 10^5^ cycles, only surface crack initiation from aluminates and pits was observed. At longer lifetimes, however, cracks mainly initiated from surface MnS inclusions or elongated stringers. Only two specimens failed from interior MnS inclusions.

#### 3.1.2 Specimens containing a small drilled hole

**FIGURE 4:**
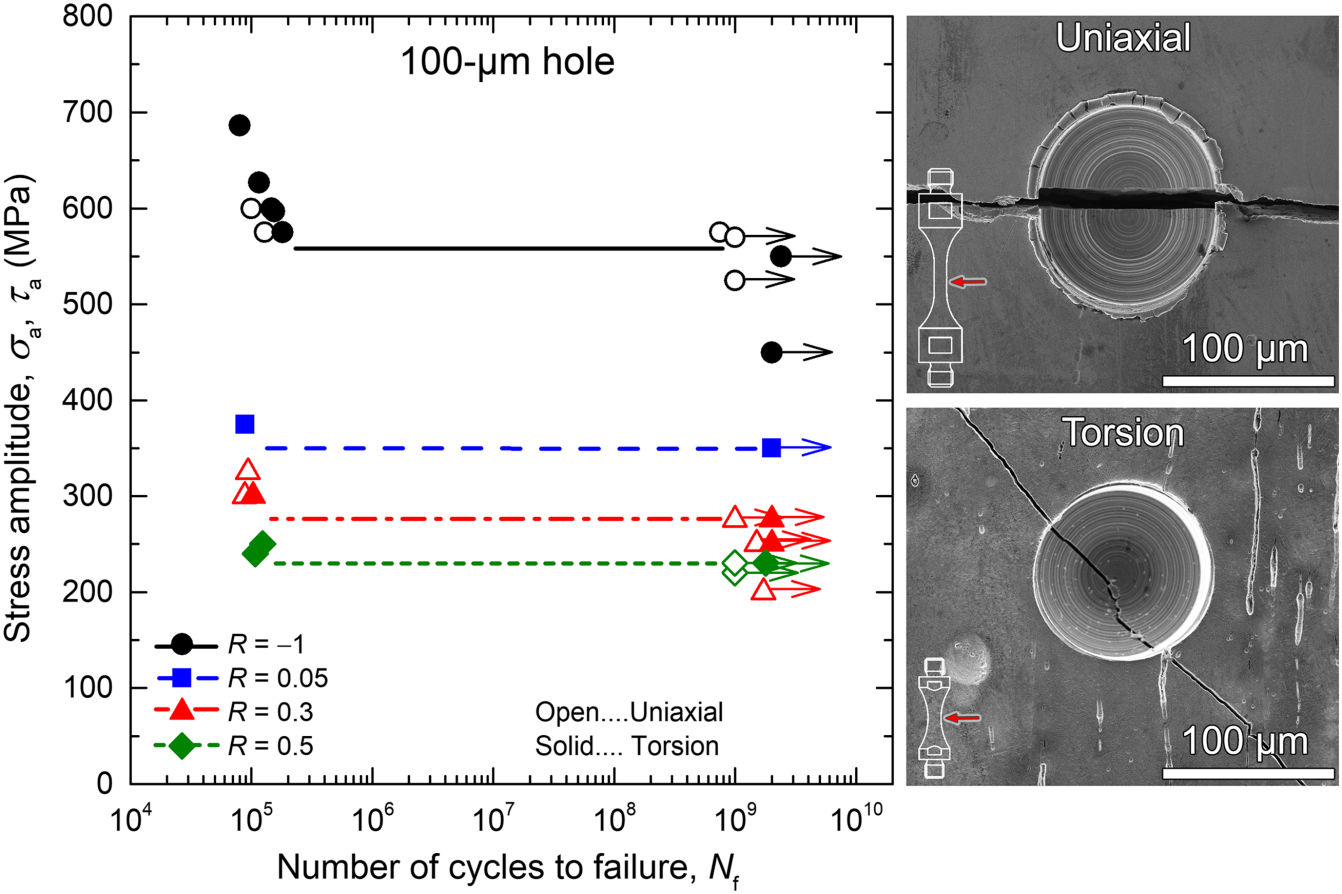
Test results with specimens containing a 100‐μm drilled hole(some data obtained under uniaxial loading at *R* = −1 were already reported in Schönbauer et al.^24^) [Colour figure can be viewed at wileyonlinelibrary.com]

Fatigue specimens containing drilled holes were tested up to at least 10^9^ cycles if no failure has occurred before then. Experiments under torsional loading were conducted at *R* = −1, 0.05, 0.3, and 0.5. Under axial loading, the fatigue limits were determined at *R* = −1 and 0.3, and two tests were performed at *R* = 0.5 without causing fracture. The results obtained with specimens containing 100‐μm holes are plotted in Figure 4, and further results with 50 and 200 μm will be presented in Kitagawa–Takahashi diagrams (Figure 12). As shown in Figure 4, identical fatigue limits were determined under uniaxial and torsional loading at *R* = −1 and 0.3. The *S–N* curves exhibit clear knee points between 1 × 10^5^ and 2 × 10^5^ cycles. Only one specimen tested under fully reversed axial loading failed in the VHCF regime, which might be explained by environmental effects: The observability of nonpropagating cracks at small surface defects after testing at stress amplitudes below the fatigue limit, as demonstrated in a prior study^24^ performed at *R* = −1, suggests that these arrested cracks can further grow after a sufficient number of load cycles in a chemically active environment (as ambient air for ultrahigh‐strength steels). This effect is most pronounced at negative load ratios since nonpropagating cracks can be hardly observed at high tensile mean stresses (where once initiated cracks tend to further propagate until failure due to a lower contribution of crack closure). This issue has been discussed in detail in Schönbauer et al.^24^

### 3.2 Fractography

The fracture surfaces of all specimens were observed using FE‐SEM. Run‐out specimens were retested at higher stress amplitudes in order to detect the most detrimental defect or the potential crack‐initiation site of each specimen. The size of each defect (inclusion, pit or grain boundary) was evaluated in terms of the square root of its projection area perpendicular to the major principal stress direction, $\sqrt{\text{area}}$, as proposed by Murakami and Endo.^19^

#### 3.2.1 Uniaxial loading

**FIGURE 5:**
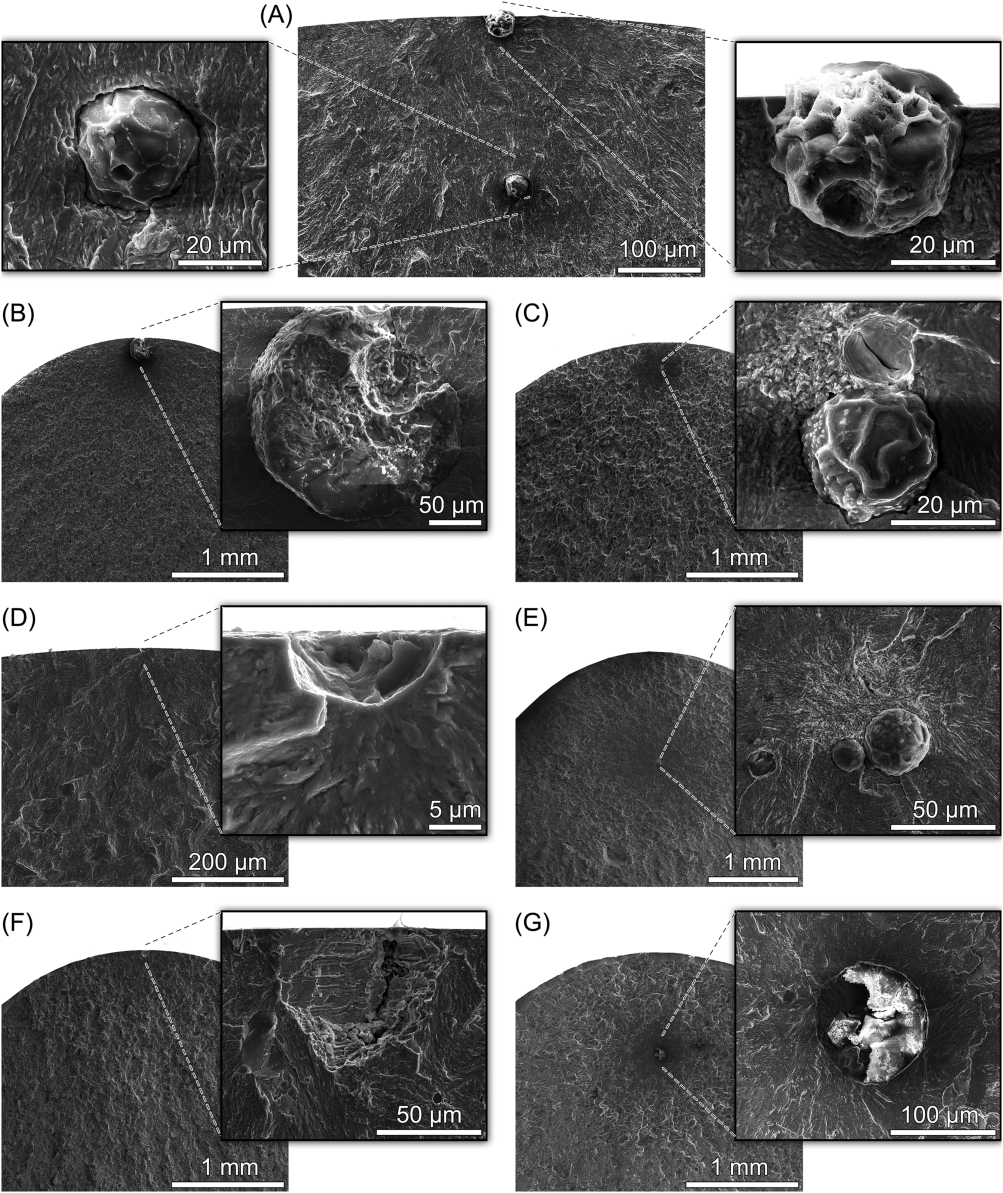
Origins of fatigue fracture under uniaxial loading (aluminate inclusions or remaining pits): (A) primary surface and secondary interior inclusion, *R* = −1, *σ*_a_ = 625 MPa, *N*_f_ = 1.60 × 10^6^ cycles, (B) surface inclusion, *R* = 0.05, *σ*_a_ = 330 MPa, *N*_f_ = 4.10 × 10^4^ cycles, (C) interior inclusions, *R* = 0.05, *σ*_a_ = 457 MPa, *N*_f_ = 2.09 × 10^8^ cycles, (D) surface pit, *R* = 0.3, *σ*_a_ = 325 MPa, *N*_f_ = 7.20 × 10^7^ cycles, (E) interior inclusions, *R* = 0.3, *σ*_a_ = 325 MPa, *N*_f_ = 1.91 × 10^9^ cycles, (F) surface pit, *R* = 0.5, *σ*_a_ = 300 MPa, *N*_f_ = 4.70 × 10^4^ cycles, and (G) interior inclusion, *R* = 0.5, *σ*_a_ = 300 MPa, *N*_f_ = 1.10 × 10^7^ cycles

For most specimens tested under uniaxial loading, nonmetallic inclusions were observed at the origin of fracture. EDX analyses suggest that these inclusions are calcium aluminates Al_2_O_3_–CaO. An increased amount of sulfur indicates that the aluminates are encapsulated in calcium sulfides. As shown in Figure 5A, two inclusions—one at the surface and the other one in the interior—were observed on the fracture surface of a specimen that failed after 1.60 × 10^6^ cycles (*R* = −1, *σ*_a_ = 625 MPa). It seems that both inclusions were crack‐initiation sites, although the surface inclusion was the dominating one (which can be easily explained by both its slightly larger size and its location at the surface). Figure 5B shows the largest inclusion ( $\sqrt{\text{area}}$ = 195 μm) observed on the fracture surface of all specimens tested; failure already occurred after *N*_f_ = 4.10 × 10^4^ cycles although the stress amplitude was relatively low (*σ*_a_ = 330 MPa, *R* = 0.05). Furthermore, inclusion clusters of two or more aluminates could be observed; see Figure 5C,D. A so‐called optically dark area^12^ (ODA)—which is often observed around crack‐initiating inclusions located in the interior—is discernible on both fracture surfaces. The location of the ODA between the inclusions and the pit (the second inclusion was found on the opposite fracture surface) shown in Figure 5C reveals the interaction of both inclusions during the crack initiation process. In Figure 5D, the ODA emanated from the two adjacent inclusions. However, the third inclusion—at a distance of about 30 μm from the others—apparently did not contribute to the fracture process. Hence, only the size of the two adjacent inclusions was used to determine the size‐parameter $\sqrt{\text{area}}$.

Surface pits could also be identified as crack‐initiation sites. The pit shown in Figure 5D (*R* = 0.3) probably originated from an inclusion that fell out during electropolishing. Similar pits have been observed for other steels if the surface of fatigue test specimens was treated similarly; see, e.g., Schönbauer et al.^22^ The morphology of the pit shown in Figure 5F (*R* = 0.5) appears slightly different, which suggests that the inclusion is located in the second fracture surface of the specimen. However, another pit was observed on the opposite site. A possible explanation for this is the detachment of an aluminate caused by the high tensile mean stress during testing at *R* = 0.5.

**FIGURE 6:**
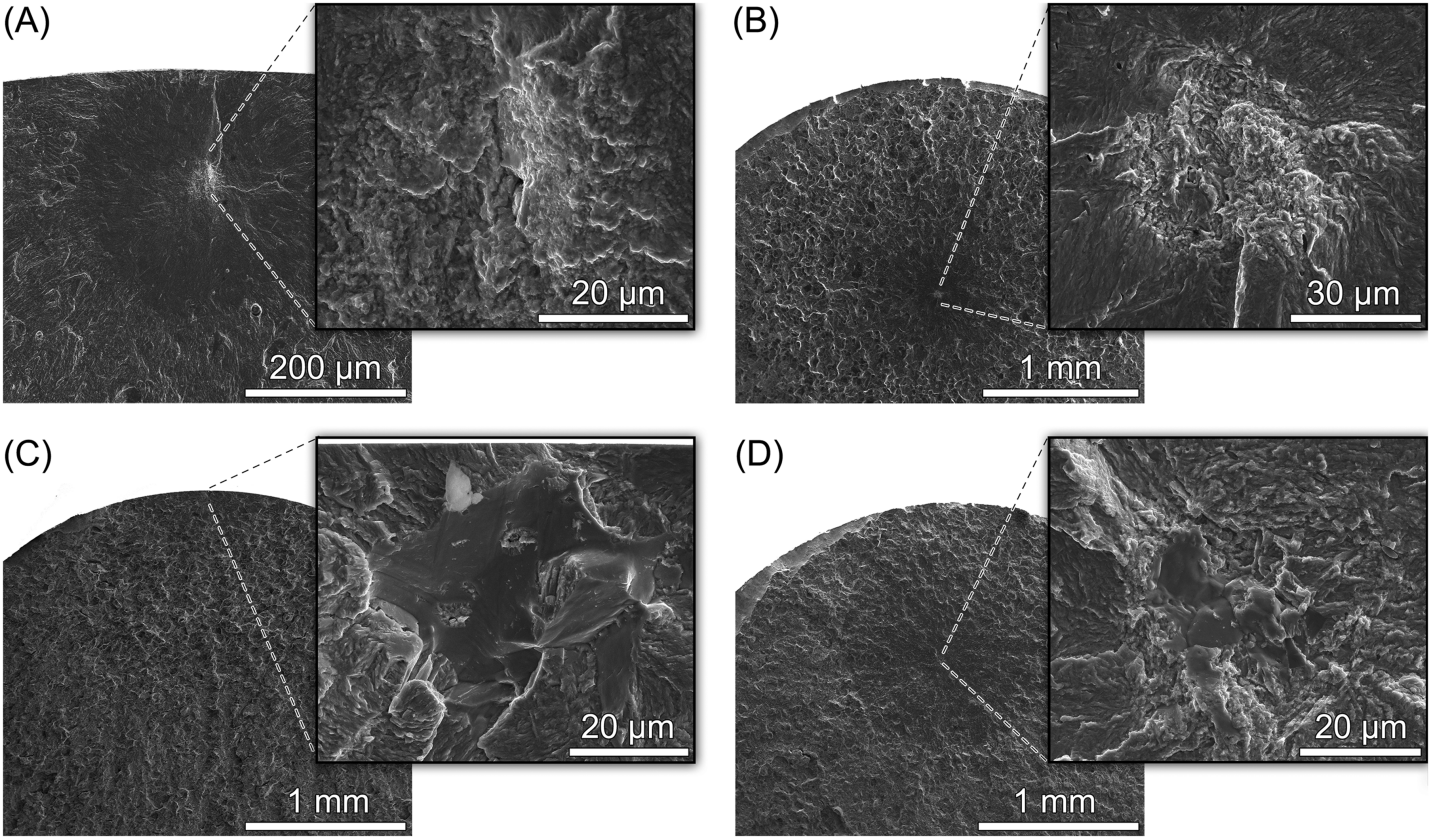
Origins of fatigue fracture under uniaxial loading (no aluminate inclusions): (A) interior failure without inclusion, *R* = −1, *σ*_a_ = 700 MPa, *N*_f_ = 1.24 × 10^9^ cycles, (B) interior without inclusion, *R* = 0.05, *σ*_a_ = 425 MPa, *N*_f_ = 8.52 × 10^9^ cycles, (C) subsurface grain boundary, *R* = 0.5, *σ*_a_ = 280 MPa, *N*_f_ = 6.04 × 10^7^ cycles, and (D) interior TiN inclusion and grain boundaries, *R* = 0.5, *σ*_a_ = 300 MPa, *N*_f_ = 3.21 × 10^8^ cycles

In Figure 6, fracture surfaces of some specimens labeled as “unknown” in Figure 3A are shown. The cause of failure could not be clearly identified. While EDX analyses at the origins of fracture shown in Figure 6A–C revealed no differences in chemical composition compared to the surrounding matrix, a small TiN inclusion could be identified for the specimen that failed from the interior after testing at *R* = 0.5 (Figure 6D). The size of this TiN inclusion ( $\sqrt{\text{area}}$ = 9 μm) was much smaller than those of aluminates ( $\sqrt{\text{area}}$ = 28–116 μm, if the outlier with a size of $\sqrt{\text{area}}$ = 195 μm shown in Figure 5B is neglected), but an intergranular facet could be observed adjacent to the inclusion. A subsurface intergranular facet could also be observed at the crack initiation site of another specimen tested at *R* = 0.5 (Figure 6C), which suggests an effect of high‐mean stresses on this fracture mode (e.g., separation of weak grain boundaries).

#### 3.2.2 Torsional loading

**FIGURE 7:**
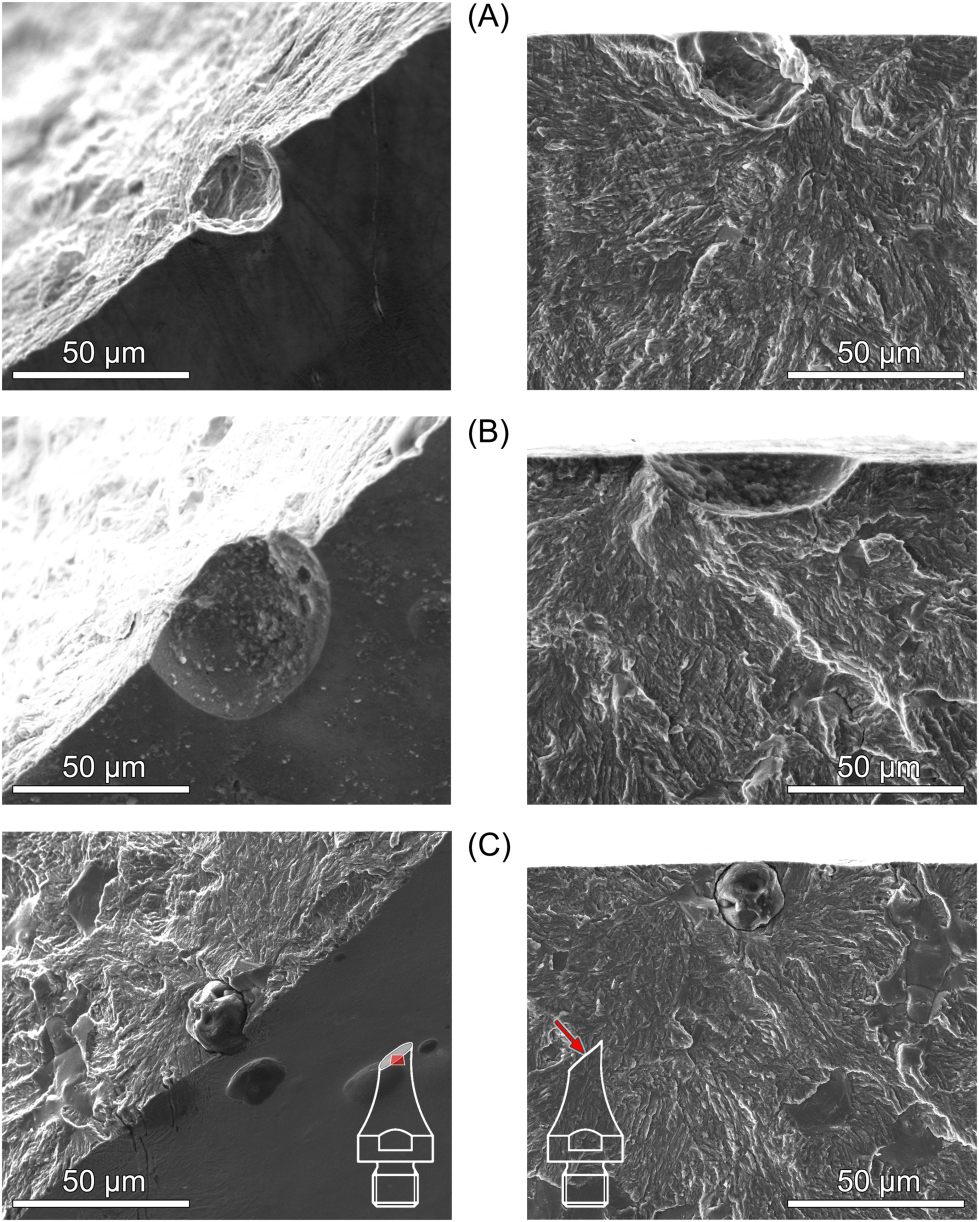
Origins of fatigue fracture under torsional loading (surface aluminate inclusions or remaining pits): (A) surface inclusion, *R* = 0.05, *τ*_a_ = 475 MPa, *N*_f_ = 8.70 × 10^4^ cycles, (B) surface pit, *R* = 0.3, *τ*_a_ = 550 MPa, *N*_f_ = 3.10 × 10^4^ cycles, and (C) surface inclusions, *R* = 0.5, *τ*_a_ = 325 MPa, *N*_f_ = 1.24 × 10^5^ cycles [Colour figure can be viewed at wileyonlinelibrary.com]

**FIGURE 8:**
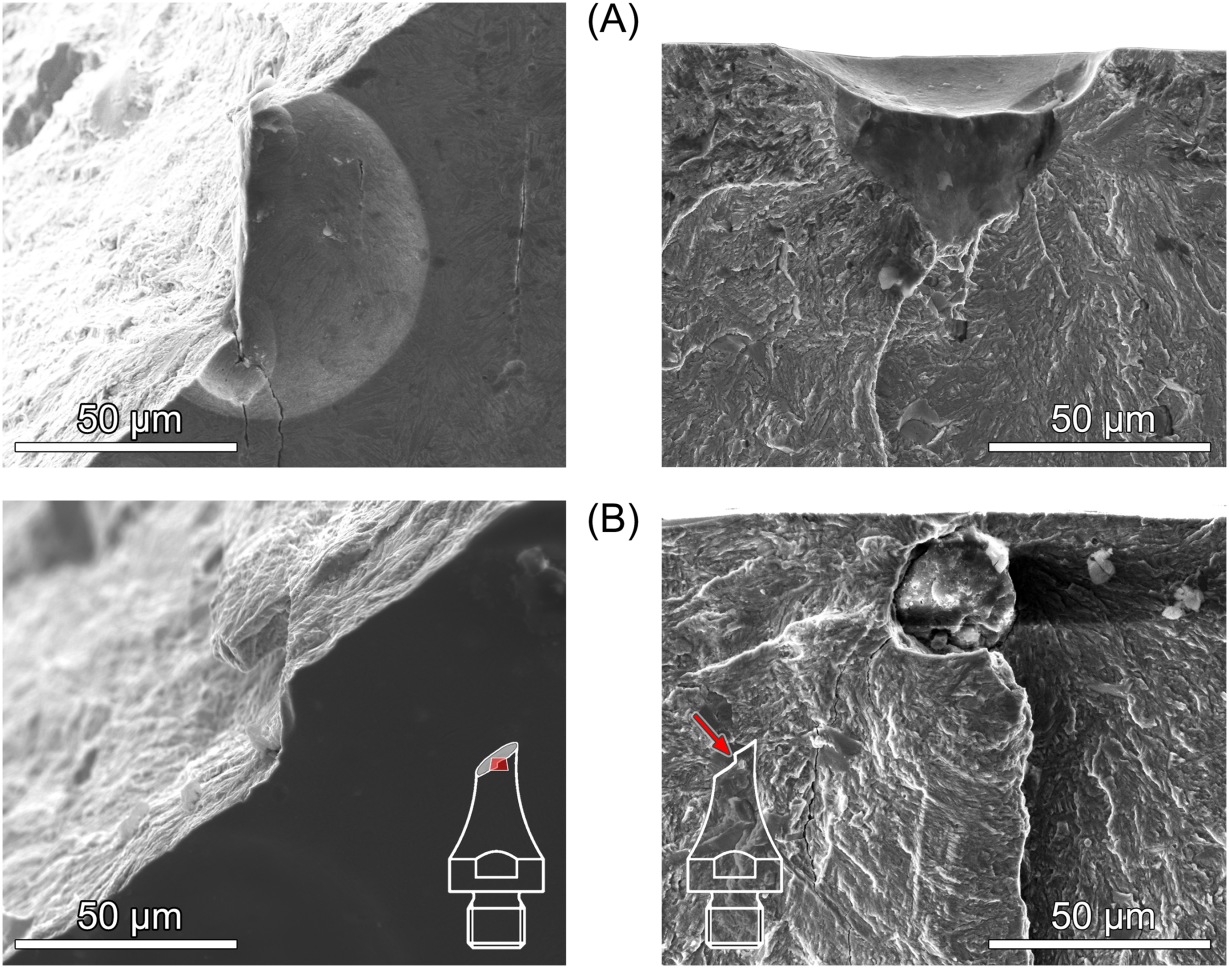
Origins of fatigue fracture under torsional loading (aluminate inclusions or remaining pits with surface shear crack): (A) surface pit with shear crack, *R* = 0.5, *τ*_a_ = 275 MPa, *N*_f_ = 1.05 × 10^5^ cycles, (B) subsurface inclusion, *R* = 0.05, *τ*_a_ = 550 MPa, *N*_f_ = 4.80 × 10^4^ cycles [Colour figure can be viewed at wileyonlinelibrary.com]

Under torsional loading, fatigue crack initiation from aluminate inclusions and pits was observed exclusively from the surface (or close to the surface) below 2 × 10^5^ cycles. Most of these cracks initiated under Mode I as shown in Figure 7. This failure mode was only observed at positive load ratios. Two specimens tested at *R* = 0.05 and 0.5 exhibit an initial Mode‐II/III crack path from the pit and the inclusion, respectively, before branching to Mode I; see Figure 8A,B. The pit shown in Figure 8A is relatively shallow; however, no evidence of shear‐mode cracking at the similarly shaped defect shown in Figure 7B was found. Therefore, it can be concluded that the driving force for Mode‐I crack initiation was insufficient—which will be further discussed in Section 3.4. The inclusion depicted in Figure 8B is located slightly below the surface, which mitigates the stress intensity of such a defect compared to a surface defect and may support initial crack propagation under shear mode.

**FIGURE 9:**
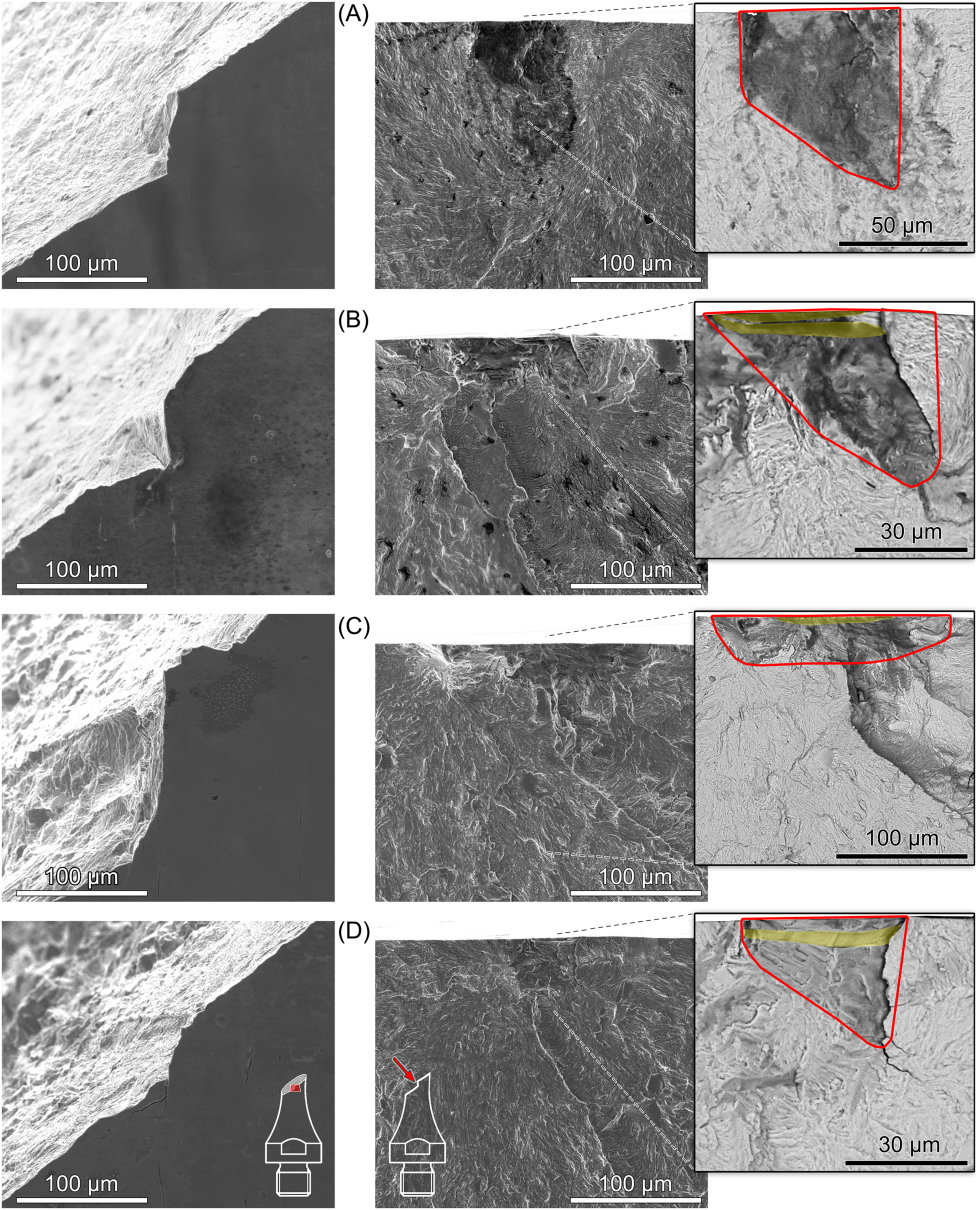
Origins of fatigue fracture under torsional loading (surface shear crack initiated at MnS inclusions): (A) *R* = −1, *τ*_a_ = 541 MPa, *N*_f_ = 1.82 × 10^8^ cycles, (B) *R* = 0.05, *τ*_a_ = 400 MPa, *N*_f_ = 4.16 × 10^7^ cycles, (C) *R* = 0.3, *τ*_a_ = 300 MPa, *N*_f_ = 3.85 × 10^9^ cycles, and (D) *R* = 0.5, *τ*_a_ = 300 MPa, *N*_f_ = 3.74 × 10^7^ cycles. MnS inclusions are marked yellowly in the high‐magnification FE‐SEM/BSE micrographs with effective sizes of shear cracks surrounded by red lines [Colour figure can be viewed at wileyonlinelibrary.com]

Between 10^6^ and 10^10^ cycles, the predominant failure mechanism under torsional loading at all load ratios was shear crack initiation followed by crack brunching and further propagation in Mode I until failure. Examples are shown in Figure 9. At most crack initiation sites, elongated MnS inclusions oriented parallel to the specimens' length direction could be identified. As depicted in Figure 9, high magnification FE‐SEM images taken by backscattered electrons (BSE) enable to distinguish the approximate shape of shear cracks. This is probably caused by an increased oxidation due to rubbing of the fracture surfaces caused by Mode II/III cracking. In these images, the effective sizes of shear cracks—used to determine the size‐parameter $\sqrt{\text{area}}$ for fracture mechanics evaluation in Section 3.4—are red‐rimmed, and the MnS inclusions were highlighted with yellow color for better discriminability. MnS inclusions could not be detected on the fracture surfaces of all specimens (see, e.g., Figure 9A). However, hollowed stringers were regularly observed at the location of surface shear cracks that may be a result of dissolution of MnS inclusions during electropolishing. Such stringers can be, for example, seen in Figure 4.

**FIGURE 10:**
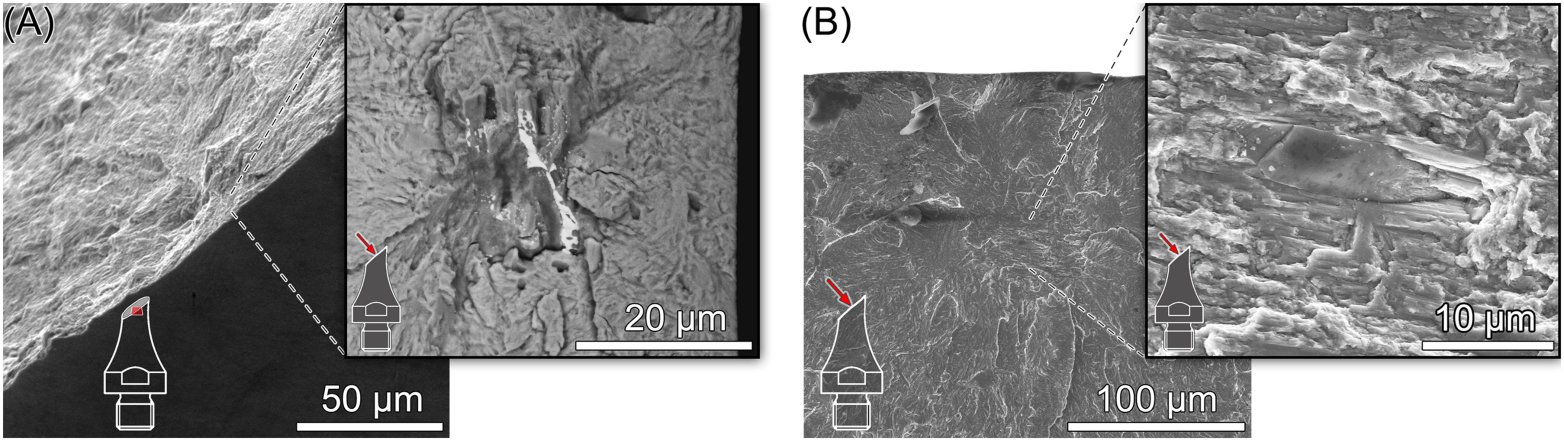
Origins of fatigue fracture under torsional loading (interior MnS inclusions): (A) *R* = 0.3, *τ*_a_ = 400 MPa, *N*_f_ = 2.88 × 10^7^ cycles, and (B) *R* = 0.5, *τ*_a_ = 325 MPa, *N*_f_ = 1.09 × 10^9^ cycles [Colour figure can be viewed at wileyonlinelibrary.com]

Failure from interior MnS inclusions took place merely in two specimens; see Figure 10. These crack‐initiating inclusions were located close to the surface (about 20 and 100 μm), which can be explained by the stress gradient effective from the surface to the interior of specimens under torsional loading. Both specimens failed in the VHCF regime.

### 3.3 Mean stress sensitivity

Murakami and Endo^18^, ^19^ proposed a simple model that allows to predict the fatigue limit in the presence of small cracks and defects. According to this model, the fatigue limit is determined by the hardness of the material (Vickers hardness, *HV*, in kgf/mm^2^) and the square root of the projection area of a defect perpendicular to the major principal stress direction ( $\sqrt{\text{area}}$ in μm). Incorporating the effect of load ratio, *R*, the fatigue limit, *σ*_w_ (in MPa), can be predicted by following equation^16^:

(1) $$\sigma_{w}=\frac{h\cdot \left(\mathrm{HV}+120\right)}{{\left(\sqrt{\text{area}}\right)}^{1/6}}\cdot {\left(\frac{1-R}{2}\right)}^{\alpha },$$

with *h* = 1.43 for surface cracks/defects and *h* = 1.56 for interior cracks/defects.

**FIGURE 11:**
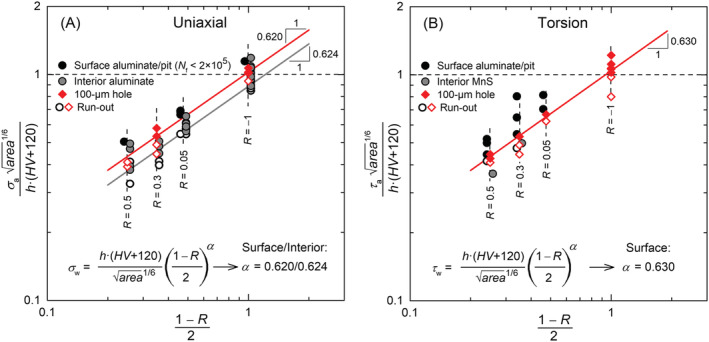
Determination of *R*‐ratio dependency under (A) uniaxial and (B) torsional loading. The values for surface and interior inclusions are slightly shifted to the left and right, respectively, on the abscissa for better discriminability [Colour figure can be viewed at wileyonlinelibrary.com]

The exponent *α* in the Walker term,^26^ ([1 − *R*]/2)^*α*^, is a material constant between 0 and 1 that quantifies the material's mean‐stress sensitivity. The original formulation of the exponent given by Walker is (1 − *γ*), which means that the material's sensitivity to mean stress increases with decreasing value of *γ*. The expression used by Murakami for Equation 1 with *α* = (1 − *γ*), in contrast, leads to higher sensitivity with increasing *α*. The value of *α* for Ck45M steel (TMP‐DQ) was determined based on the *S*–*N* data presented in Figures 3 and 4. According to the method proposed in Schönbauer et al.,^22^ the test results were plotted in a double‐logarithmic diagram with the abscissa (1 − *R*)/2 and the ordinate (*σ*_a_· $\sqrt{\text{area}}$ ^1/6^)/(*h*·(*HV* + 120)) according to Equation 1, as shown in Figure 11. Power curve fits through the lowest solid symbols (open symbols mark run‐out specimens) at each load ratios reveal the value of *α*. The regression lines adequately fit the data for 100‐μm holes, and interior aluminate inclusions with coefficients of determination of *r* ^2^ > 0.997. Values of *α* = 0.620 and 0.630 were determined for drilled holes under uniaxial and torsional loading, respectively, and *α* = 0.624 for interior aluminate inclusions under uniaxial loading. For the sake of conservatism, *α* = 0.63 will be used in the following.

The mean‐stress sensitivity under torsional loading was evaluated by assuming a ratio of torsional and uniaxial fatigue limit of *τ*_w_/*σ*_w_ = 1 (which does not affect determination of *α*); see Figure 11B. The dashed, horizontal lines plotted in Figure 11 represent the predicted fatigue limit at *R* = −1 calculated with Equation 1. All data points representing failure at fully reversed loading with specimens containing 100‐μm holes lie above this line. Further, all data points marking failure from surface aluminates or pits (black solid symbols) are above the regression line determined with 100‐μm holes. This demonstrates the applicability of Equation 1 for surface defects—for both axial and torsional loading with *τ*_w_ = *σ*_w_.

As shown in Figure 4, all specimens containing small drilled holes either failed below *N*_f_ < 2 × 10^5^ cycles or did not fail at all (with the exemption of one specimen that failed in the VHCF regime, see Section 3.1.2). Therefore, only data with failure below *N*_f_ < 2 × 10^5^ cycles were plotted in Figure 11A if fracture emanated from a nonmetallic inclusion or a pit located at the surface. Failure at longer lifetimes can be explained by superimposed environmental effects, which may reduce the conventional fatigue limit of ultrahigh‐strength steels. An important fact in this regard is that—in contrast to a drilled hole—a nonmetallic inclusion typically exhibits locally very small notch‐root radii (due to its irregular shape) inducing high stress concentrations that enable crack initiation even at stress amplitudes significantly below the fatigue limit.^27^ However, due to build‐up of crack closure, these cracks become nonpropagating. With increasing root radius (i.e., decreasing stress concentration), the threshold for crack initiation approaches the threshold for crack propagation, thus making nonpropagating cracks—below the fatigue limit—a rare phenomenon. Since degrading environmental effects such as air humidity, can be regarded more harmful to crack propagation (i.e., elongation of a crack that would arrest in an inert environment) than to crack initiation (e.g., formation of a corrosion pit where a crack can initiate), VHCF surface failure can be considered as the consequence of “non”‐propagating cracks being driven to further propagate by environmental influences. A corroboration of this hypothesis is the nonexistence of failure from surface aluminates (and drilled holes) beyond 2 × 10^5^ cycles at positive load ratios: Nonpropagating cracks can be generally rarely observed in the presence of high mean stresses due to the diminishing effect of crack closure. This is of course only true for Mode‐I cracks. Mean shear loads under torsional loading do not open a Mode‐II or ‐III crack and therefore have no pronounced effect on crack closure. Hence, the observability of VHCF failure from the surface at all *R*‐ratios for smooth specimen under torsional loading—where cracks initiate under Mode‐II/III from elongated MnS stringers—may be caused by environmental effects.

It should be further noted that, strictly speaking, Equation 1 is not applicable for 100‐μm holes because the size of these defects, with respect to the size dependency of the threshold stress intensity factor range, is too large. This means that the fatigue limit should be determined by the (constant, i.e., size‐independent) large‐defect threshold, Δ*K*_th,ld_, rather than the size‐dependent threshold stress intensity factor range (from which Equation 1 is derived, see Murakami^16^). However, due to the constant size of 100‐μm holes, it is not necessary to distinguish between small and large defects when determining the value of *α*. The differentiation between small and large defects will be discussed in more detail in the next section.

### 3.4 Fracture mechanics evaluation

As demonstrated above, the fatigue limit under uniaxial and torsional loading condition is identical, i.e., *τ*_w_/*σ*_w_ = 1, provided that failure mechanism (Mode‐I cracking) and defect sizes are the same. In the presence of small defects, *σ*_w_ and *τ*_w_ can then be calculated according to the $\sqrt{\text{area}}$‐parameter model and using *α* = 0.63:

(2) $$\sigma_{w}=\tau_{w}=\frac{h\cdot \left(\mathrm{HV}+120\right)}{{\left(\sqrt{\text{area}}\right)}^{1/6}}\cdot {\left(\frac{1-R}{2}\right)}^{0.63}\text{for}\;\sqrt{\text{area}}<{\sqrt{\text{area}}}_{\text{trans}},$$

with *h* = 1.43 for surface defects and *h* = 1.56 for interior defects and the Vickers hardness *HV* = 717.

The validity of Equation 2, however, is limited to small defects, i.e., to defects smaller than the transition size ${\sqrt{\text{area}}}_{\text{trans}}$—which is a material constant as demonstrated in prior studies.^11^, ^28^ For defects larger than ${\sqrt{\text{area}}}_{\text{trans}}$, the threshold stress intensity factor range for large defects (referring to the long‐crack threshold), Δ*K*_th,ld_, must be applied. The value of Δ*K*_th,ld_ for the investigated ultrahigh‐strength steel has been determined under fully reversed uniaxial loading in a previous study^16^ as ${\left.∆K_{\mathrm{th},\mathrm{ld}}\right|}_{R=-1}$ = 12 MPa $\sqrt{m}$. For defects larger than ${\sqrt{\text{area}}}_{\text{trans}}$, following prediction equation can be applied^28^:

(3) $$\sigma_{w,\mathrm{ld}}=\tau_{w,\mathrm{ld}}=\frac{i\cdot {\left.∆K_{\mathrm{th},\mathrm{ld}}\right|}_{R=-1}}{{\left(\sqrt{\text{area}}\right)}^{1/2}}\cdot {\left(\frac{1-R}{2}\right)}^{0.63}\text{for}\;\sqrt{\text{area}}\geq {\sqrt{\text{area}}}_{\text{trans}},$$

with *i* = 434 for surface defects and *i* = 564 for interior defects ( $\sqrt{\text{area}}$ is in μm).

The transition size between small and large defects, ${\sqrt{\text{area}}}_{\text{trans}}$ (in μm), is independent of load ratio and can be calculated by^28^:

(4) $${\sqrt{\text{area}}}_{\text{trans}}={\left(\frac{{\left.∆K_{\mathrm{th},\mathrm{ld}}\right|}_{R=-1}}{g\cdot \left(\mathrm{HV}+120\right)}\right)}^{3},$$

with *g* = 3.3 × 10^−3^ for surface defects and *g* = 2.77 × 10^−3^ for interior defects. According to Equation 4, the transition sizes is ${\sqrt{\text{area}}}_{\text{trans},s}$ = 82 μm for surface defects and ${\sqrt{\text{area}}}_{\text{trans},i}$ = 139 μm for interior defects. Derivations of Equations 1–4 can be found in Schönbauer et al.^11^, ^24^, ^28^

**FIGURE 12:**
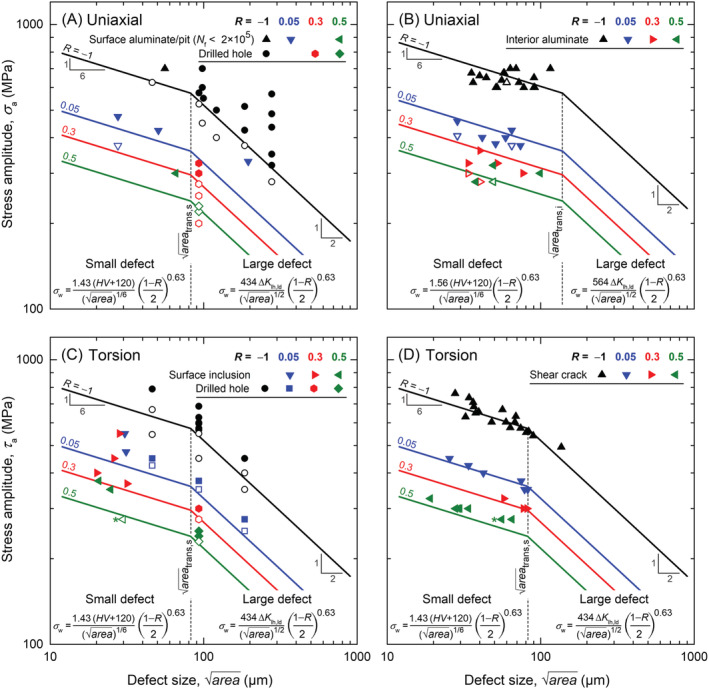
Kitagawa–Takahashi diagrams for (A) uniaxial loading with failure from surface inclusions and drilled holes, (B) uniaxial loading with failure from interior inclusions (most data obtained at *R* = −1 were already reported in Schönbauer et al.^24^), and (C) torsional loading with failure from surface inclusions and drilled holes. In (D), the relation between shear crack sizes observed on the fracture surfaces and shear stress amplitudes is given. Run‐out specimens are marked with open symbols [Colour figure can be viewed at wileyonlinelibrary.com]

The experimental results are compared with the predictions according to Equations 2 and 3 in Kitagawa–Takahashi diagrams. In Figure 12A,C, data obtained with specimens containing small drilled holes clearly show that the fatigue limit in the presence of surface defects larger than the transition size of ${\sqrt{\text{area}}}_{\text{trans}}$ = 82 μm can be predicted with Equation 3, both under uniaxial and torsional loading condition. Whereas, for smaller holes (*d* = 50 μm) as well as surface aluminates and pits, Equation 2 renders useful results. As mentioned in Section 3.3, the size of 100‐μm holes with $\sqrt{\text{area}}$ = 93 μm is slightly above the transition size, and therefore, Equation 2 is technically speaking not applicable. However, due to the constant defect size, the load‐ratio dependency (i.e., the value of *α*) was appropriately determined.

Although prediction Equation 2 is slightly nonconservative for failure from interior aluminate inclusions, as shown in Figure 12B and further discussed below, the load ratio dependency is appropriately considered.

Most interesting results are depicted in Figure 12D, where the sizes of shear cracks starting from MnS stringers are plotted versus the shear stress amplitudes. As shown in Figure 9, the sizes of surface shear cracks could be measured from FE‐SEM/BSE fractographs. These crack sizes can be obviously correlated with the fatigue limit, *τ*_w_. Recent investigations by Endo and Yanase^29^ and Schönbauer et al.^17^ demonstrated that the torsional fatigue limit is determined by the threshold condition for Mode‐I crack propagation. In the case of shear‐crack initiation, this means that the incipient Mode‐II/III crack must exceed a critical size before branching and subsequent propagation in Mode I is possible.^30^ Equations 2 and 3 for small and large cracks/defects have been derived from size‐dependent^16^ and constant^28^ threshold stress intensity factors for Mode‐I cracks, respectively. Hence, the correlation between the predicted fatigue limit and the shear crack size as shown in Figure 12D corroborates this assumption.

As shown in Figure 8A, a smooth specimen tested at *τ*_a_ = 275 MPa (*R* = 0.5) failed due to shear mode cracking at the base of a surface pit. Plotting only the pit size excluding (datapoint marked with * in Figure 12C) and including the shear crack (datapoint marked with * in Figure 12D) in the Kitagawa–Takahashi diagrams reveals that the pit was too small to initiate a Mode‐I crack. However, the driving force for shear‐mode cracking was obviously sufficient, and crack brunching followed by Mode‐I crack growth occurred after exceeding the threshold value expressed by Equation 2.

**FIGURE 13:**
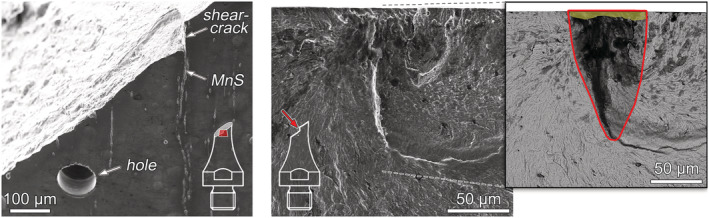
Origin of fatigue fracture (shear cracking at an MnS inclusion) under torsional loading (*R* = −1, *τ*_a_ = 560 MPa, *N*_f_ = 1.40 × 10^7^ cycles) of a specimen containing a 100‐μm hole. The MnS inclusion is yellowish marked in the high‐magnification FE‐SEM/BSE micrograph with the effective size of the shear crack surrounded by a red line [Colour figure can be viewed at wileyonlinelibrary.com]

Another interesting observation was made with a torsional specimen containing a 100‐μm drilled hole tested at the fatigue limit (*τ*_w_ = 560 MPa, *R* = −1). As shown in Figure 13, failure occurred in some distance to the hole from a MnS stringer. The size of the shear crack measured from the FE‐SEM/BSE fractograph $(\sqrt{\text{area}}$ = 83 μm) is slightly smaller than the hole size ( $\sqrt{\text{area}}$ = 93 μm). This means that the hole should be more detrimental from a fracture mechanics point of view. However, considering the morphology of a sulfide stringer with a small notch root radius, it is reasonable that shear cracks can easily initiate—even at stresses below the fatigue limit. The fatigue limit predicted by Equation 3 considering the shear‐crack size of $\sqrt{\text{area}}$ = 83 μm is *τ*_w_ = 570 MPa—which is 2% above the applied shear stress amplitude—is again in good accordance with the evaluation given above (Figure 12D).

### 3.5 Fatigue limit prediction

**FIGURE 14:**
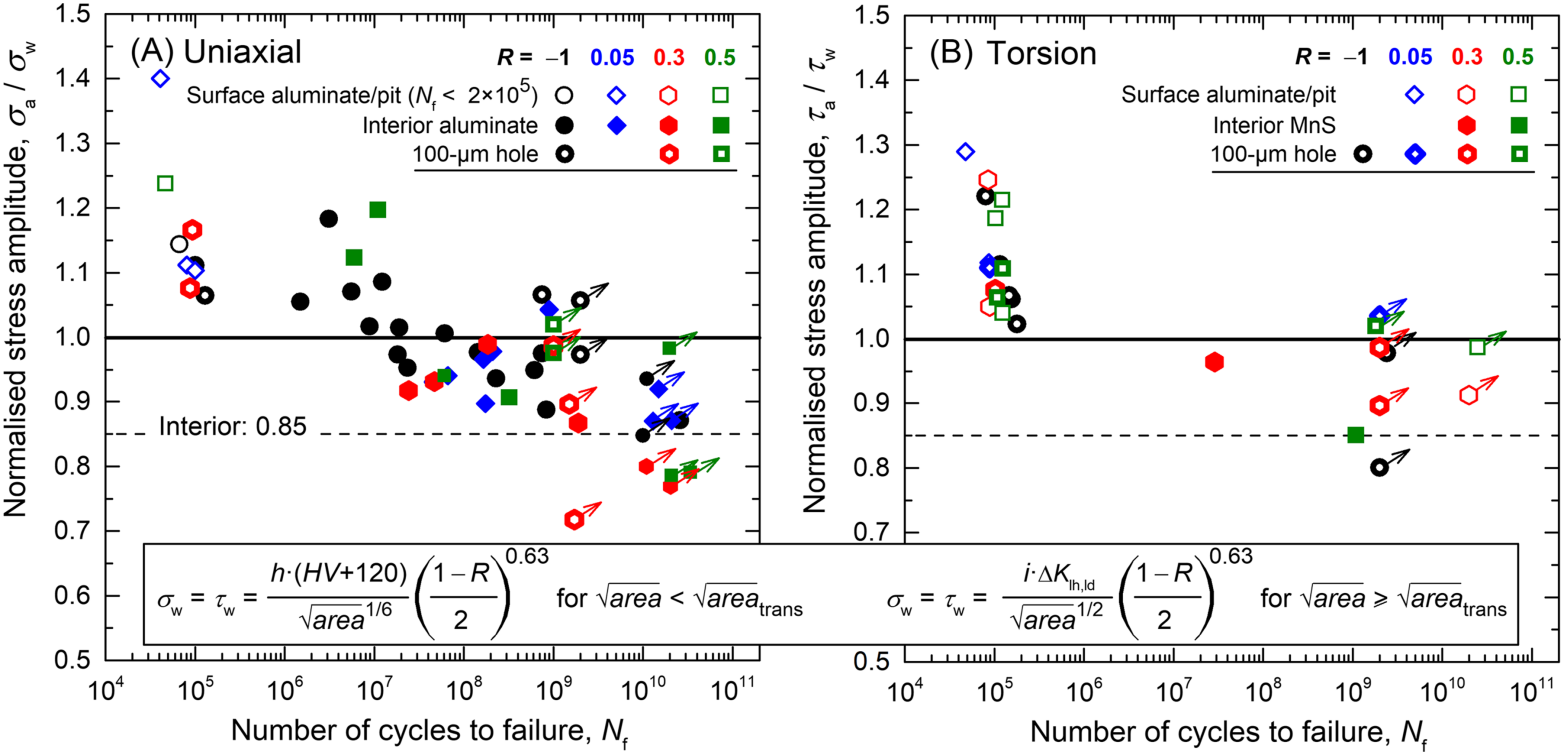
Normalized stress amplitude, *σ*_a_/*σ*_w_ and *τ*_a_/*τ*_w_, versus number of cycles to failure for (A) uniaxial and (B) torsional loading. Run‐out specimens are marked with arrows [Colour figure can be viewed at wileyonlinelibrary.com]

In Figure 14, the results of specimens that failed from surface and interior aluminates, 100‐μm holes and—as observed under torsional loading—from interior MnS inclusions are plotted in normalized *S*–*N* diagrams. In these diagrams, the stress amplitude divided by the predicted fatigue limit is shown on the ordinate.^31^ Each specimen possesses its own fatigue limit, which can be calculated according to the size of the detrimental defect—determined after fatigue fracture by fractographic observation—and Equations 2 and 3. If the prediction is accurate, no data point representing failure should be below a value of *σ*_a_/*σ*_w_ = 1 and *τ*_a_/*τ*_w_ = 1.

#### 3.5.1 Failure from the surface

The prediction is obviously accurate for all test results where fracture initiated from the surface. Also, the scatter of normalized *S*–*N* curves is very low, which demonstrates that the size of defects and the threshold stress intensity factor range is taken properly into account.

In order to disregard the time‐ (and cycle‐) dependent effect of environmental influences, fracture from surface aluminates and pits are plotted in Figure 12A (and Figure 14A) exclusively for numbers of cycles to failure below 2 × 10^5^ cycles. However, at fully reversed tension–compression loading, failure from the surface was also observed in the VHCF regime (see Figure 3A). These data were evaluated in a previous study,^24^ where it has been shown that fatigue failure occurred even at *σ*_a_/*σ*_w_ = 0.73 from a small surface pit at *N*_f_ = 6.48 × 10^10^ cycles. At positive load ratios, failure from surface inclusions and pits were not identified, which can be explained by the absence of nonpropagating cracks at stress amplitudes below the fatigue limit at high tensile mean stresses. This, as well as the possibility of shear crack initiation under torsional loading below the fatigue limit even at positive mean shear loads, has been already discussed in Section 3.3. In summary, it can be concluded that Equations 2 and 3 serve very well to predict the fatigue limit in the presence of surface aluminate inclusions and pits under both axial and torsional loading condition. The only exemption is fully reversed tension–compression loading where the fatigue limit is reduced by around 30% compared to the prediction. Equations 2 and 3 are further not applicable for sulfide stringers where shear cracks can easily initiate at shear stresses amplitudes (at all load ratios) that are significantly below *τ*_w_.

#### 3.5.2 Failure from the interior

By considering the size of interior inclusions and the size‐dependent threshold stress intensity factor range for small defects (expressed by Equation 2), the scatter of normalized *S*–*N* curves is significantly lower than of conventional *S*–*N* curves; see Figures 3 and Figure 14. However, Equation 2 considerably overestimates the fatigue limit (Equation 3 could not be applied since the size of interior inclusions were below the transition size ${\sqrt{\text{area}}}_{\text{trans},i}$ = 139 μm): With tests performed up to more than 10^10^ cycles, failure even occurred at *σ*_a_/*σ*_w_ = 0.87 and *τ*_a_/*τ*_w_ = 0.85 under uniaxial and torsional loading, respectively. Although only two torsional specimens failed due to crack initiation from the interior, the results are comparable with those obtained under uniaxial loading. It should be noted that the shear stress amplitude, *τ*_a_, in the interior of torsional test specimens is reduced due to the stress gradient under torsional loading. For the present evaluation in Figure 14B, the local stress amplitude at the position of crack‐initiating MnS inclusions is used.

Nonconservative results obtained for interior fracture in the VHCF regime with respect to Equation 2 were also reported for other ultrahigh‐strength steels,^16^, ^32^, ^33^ and the reduction in fatigue strength is associated with the occurrence of a so‐called optically dark area (ODA),^12^ a fine‐granularly appearing area on the fracture surface around interior inclusion that appears dark when observed with light microscopy. Examples of ODAs can be seen in Figures 5C,E and 6B,D. A detailed discussion on the role of ODAs has been given in a prior study on the VHCF properties of the investigated ultrahigh‐strength steel.^24^ Concerning the predictability of the fatigue strength in the VHCF regime for the investigated Ck45M steel (TMP‐DQ) with respect to interior crack initiation, it is concluded that Equation 2 overestimates the fatigue limit at 10^10^ cycles by about 15% and hence can be modified as follows:

(5) $$\sigma_{w}=\tau_{w}=0.85\cdot \frac{1.56\cdot \left(\mathrm{HV}+120\right)}{{\left(\sqrt{\text{area}}\right)}^{1/6}}\cdot {\left(\frac{1-R}{2}\right)}^{0.63}\text{for}\;\sqrt{\text{area}}<{\sqrt{\text{area}}}_{\text{trans}}.$$

#### 3.5.3 Comparison between uniaxial and torsional fatigue strength

Fracture mechanics evaluation of the experimental results has shown that the fatigue limits under uniaxial and torsional loading coincide, provided that the same crack initiation mechanism is prevalent: If crack initiation at defects (or shear cracks) above the fatigue limit is in Mode I, *τ*_w_/*σ*_w_ = 1. This is illustratively demonstrated in Figure 4 with specimens containing 100‐μm holes. Experimental as well as analytical investigations on the ratio of torsional and uniaxial fatigue limit in the presence of small notches and detrimental defects, in contrast gave values between *τ*_w_/*σ*_w_ = 0.75 and 0.86.^34^, ^35^, ^36^, ^37^ However, these studies were performed with steels possessing significantly lower strength compared to the investigated Ck45M steel (TMP‐DQ). This suggests that the ratio *τ*_w_/*σ*_w_ depends on the material's ultimate strength. First results of ongoing tests with ultrahigh‐strength bearing steel 52100 seem to support this trend. The convergence of uniaxial and torsional fatigue limit with increasing material strength might be explained by changes in plastic zone sizes for Mode‐I cracks. In principle, the transverse stress occurring under torsional loading only slightly increases the plastic zone size of a Mode‐I crack compared to uniaxial loading.^38^, ^39^ However, this only applies if the nominal stress is well below the yield stress—as in the case of stresses close to the fatigue limit of ultrahigh‐strength steels, even at higher load ratios. In contrast, the plastic zone size is significantly increased in the presence of a T‐stress if the nominal stress approaches the yield strength^39^ and thus may have a contribution to a fatigue strength reduction under torsional loading for lower‐strength steels.

As shown in Section 3.3, not only the fatigue limit (under the mentioned restrictions) but also the mean‐stress sensitivity—expressed by the exponent *α* in the Walker term—is the same for uniaxial and torsional loading conditions. This is demonstrated for the first time with an ultrahigh‐strength steel, and further investigations are necessary to reveal if this is valid for other materials. Karr et al.^23^ recently published a value of *α* = 0.550 for a high‐strength spring steel under torsional loading. This is in good agreement with the trend of increasing values of *α* with increasing strength as shown in several studies under uniaxial loading.^2^, ^4^, ^11^, ^22^, ^40^, ^41^

## 4 CONCLUSIONS

Ultrasonic fatigue tests with ultrahigh‐strength Ck45M steel processed by thermomechanical rolling and subsequent direct quenching were performed under uniaxial and torsional loading at different load ratios (*R* = −1, 0.05, 0.3, and 0.5). Smooth specimens as well as specimens containing small drilled holes were used to determine the fatigue strength in the high and VHCF regime with tests up to more than 10^10^ cycles. Fracture origins were identified using scanning electron microscopy. Following results were obtained:

1. Under uniaxial and torsional loading, cracks initiated exclusively from surface aluminates or pits when the fatigue lifetime was below 2 × 10^5^ cycles. Crack initiation at interior aluminates was the prevailing failure mechanism under uniaxial loading at higher number of cycles. At tension–compression loading, VHCF failure was also observed from the surface. Failure under torsional loading above 10^6^ cycles was dominated by shear crack initiation at elongated sulfide stringers at all load ratios. Two specimens failed from the interior in the VHCF regime under torsional loading at *R* = 0.3 and 0.5. In contrast to smooth specimens, clear fatigue limits with knee‐points below 2 × 10^5^ cycles could be determined with specimens containing artificial surface defects.
2. The fatigue limit is equal under uniaxial and torsional loading, *σ*_w_ and *τ*_w_, respectively, when the fracture mechanism is Mode‐I crack initiation at a defect. This is in contrast to prior findings where *τ*_w_/*σ*_w_ ≈ 0.85 in the presence of detrimental defects. A possible explanation for this deviation is the extremely high strength of the investigated steel.
3. The fatigue limit under uniaxial and torsional loading in the presence of surface and interior defects smaller than ${\sqrt{\text{area}}}_{\text{trans},s}$ = 82 μm and ${\sqrt{\text{area}}}_{\text{trans},i}$ = 139 μm, respectively, can be predicted using the $\sqrt{\text{area}}$‐parameter model. For larger defects, the fatigue limit can be correlated with a constant threshold‐stress intensity factor range. The prediction is highly accurate for surface defects such as aluminate inclusions or pits and drilled holes if corrosive influences of ambient air are neglected. For failure from interior inclusions, the determined fatigue limit at approximately 10^10^ is approximately 15% lower than predicted.
4. The sizes of shear cracks that initiated from elongated sulfide inclusions under torsional loading could be correlated with the predicted fatigue limits similar to aluminate inclusions and drilled holes. This verifies that the threshold condition for Mode‐I crack propagation after branching from shear cracks determines the torsional fatigue limit for this fracture mode.
5. The mean‐stress sensitivity is identical for uniaxial and torsional loading and can be expressed by $\sigma_{w}\left(R\right)={\left.\sigma_{w}\right|}_{R=-1}\cdot {\left(\frac{1-R}{2}\right)}^{0.63}$ and $\tau_{w}\left(R\right)={\left.\tau_{w}\right|}_{R=-1}\cdot {\left(\frac{1-R}{2}\right)}^{0.63}$.

## AUTHOR CONTRIBUTIONS

Bernd M. Schönbauer performed the conceptualization (lead), funding acquisition (equal), investigation (lead), validation (equal), methodology (lead), formal analysis (lead), supervision (lead), project administration (equal), writing—original draft preparation (lead), visualization (lead), and review and editing (equal). Sumit Ghosh performed the investigation (supporting) and review and editing (equal). Ulrike Karr performed the investigation (supporting) and review and editing (equal). Sakari Pallaspuro performed the investigation (supporting) and review and editing (equal). Jukka Kömi performed the resources (equal), funding acquisition (equal), and review and editing (equal). Tero Frondelius performed the project administration (equal) and review and editing (equal). Herwig Mayer performed the validation (equal), resources (equal), and review and editing (equal).

## Data Availability

The data that support the finding of this study are included within the paper.
